# Supplementary material for: Bifidobacteria-Fermented Red Ginseng and Its Constituents Ginsenoside Rd and Protopanaxatriol Alleviate Anxiety/Depression in Mice by the Amelioration of Gut Dysbiosis
Source: Nutrients. 2020 Mar 26;12(4):901. doi: 10.3390/nu12040901 (PMC7230967; doi:10.3390/nu12040901)
Supplement: Supplementary file 1 [file nutrients-12-00901-s001.pdf]

[Supplement]

Bifidobacteria-fermented red ginseng and its constituents ginsenoside Rd and protopanaxatriol alleviate anxiety/depression in mice by the amelioration of gut dysbiosis

[Figure S1]

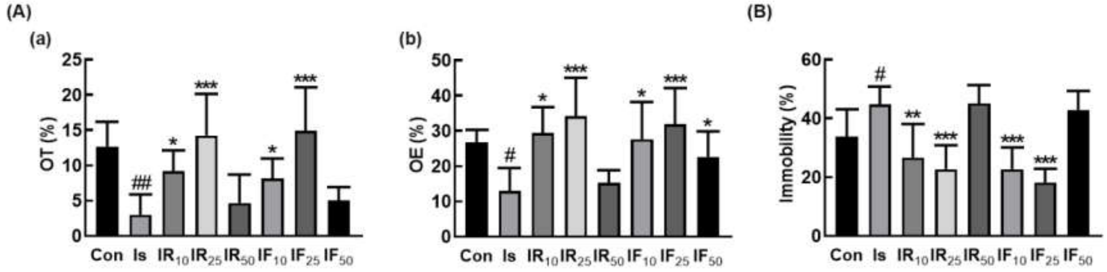

Figure S1. Oral administration of RG and fRG alleviated immobilization stress (IS)-induced anxiety/depression in mice. Effects on anxiety/depression-like behaviors in elevated plus maze (A: (a), time spent in the open arms [OT]; (b), open arm entries [OE]) and forced swimming test (B). Mice were exposed to IS. Test agents (Is, vehicle [saline]; IR_{10}, 10 mg/kg/day of RG; IR_{25}, 25 mg/kg/day of RG; IR_{50}, 50 mg/kg/day of RG; IF_{10}, 10 mg/kg/day of fRG; IF_{25}, 25 mg/kg/day of fRG; IF_{50}, 50 mg/kg/day of fRG) were gavaged daily for 5 days from 24 h after the final IS exposure. Normal control group (Con), not exposed to IS, was treated with saline instead of test agents. Data values were indicated as means ± SD (n = 6). $^{\#}p < 0.05$ vs. Con group. $^{\#}p < 0.05$ vs. Con group. $^{\#\#\#}p < 0.05$ vs. Con group. $^{*}p < 0.05$ vs. IS group. $^{***}p < 0.001$ vs. IS group.

[Figure S2]

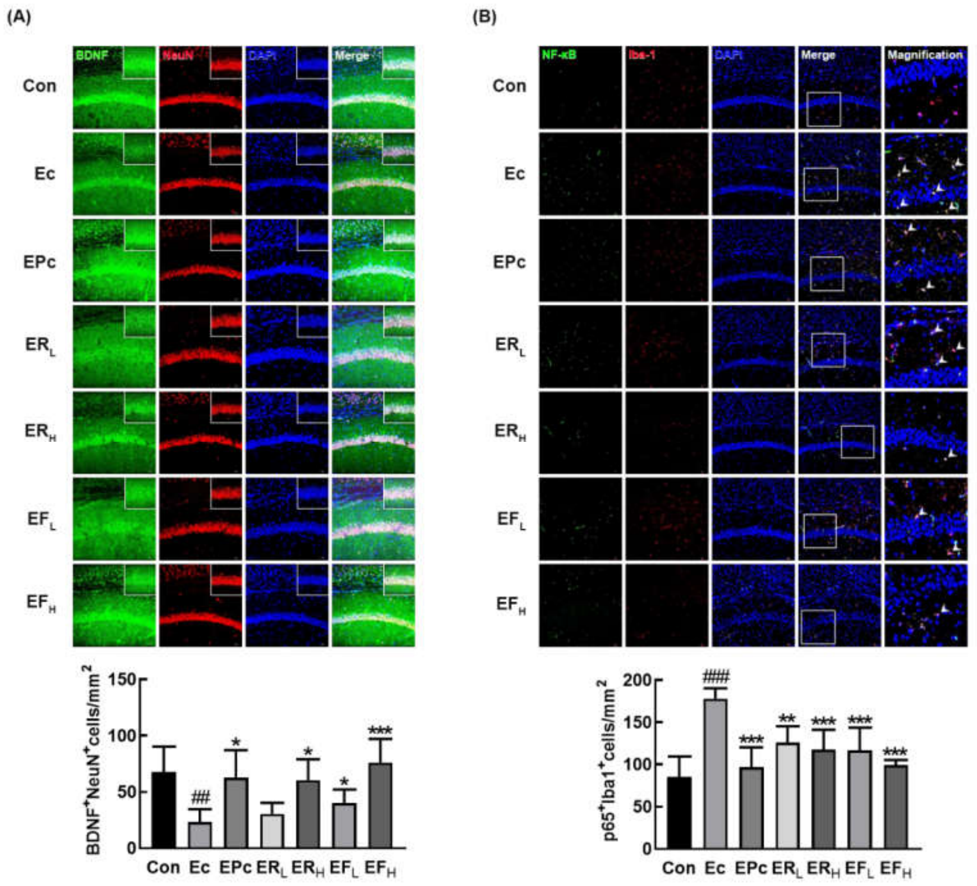

Figure S2. Effects of RG and fRG on the infiltration of BDNF$^+$/NeuN$^+$ (H) and NF-κB$^+$/Iba1$^+$ cells (I) into the CA1 region of hippocampus of *Escherichia coli* K1-exposed mice. Mice were exposed to *Escherichia coli* K1 (1×10^9 CFU/mouse/day) and test agents (Ec, vehicle [saline]; EPc, 1 mg/kg/day of buspirone; ER_L, 10 mg/kg/day of RG; ER_H, 25 mg/kg/day of RG; EF_L, 10 mg/kg/day of fRG; EF_H, 25 mg/kg/day of fRG) were gavaged (for vehicle, RG, and fRG) or intraperitoneally injected (for buspirone) daily for 5 days. Normal control group (Con), not exposed to EC, was treated with saline instead of test agents. Data values were indicated as mean ± SD (n = 6). $^{##}p < 0.01$ vs. Con group. $^{###}p < 0.05$ vs. Con group. $^{*}p < 0.05$ vs. Ec group. $^{***}p < 0.001$ vs. Ec group.

[Figure S3]

(A) (B)

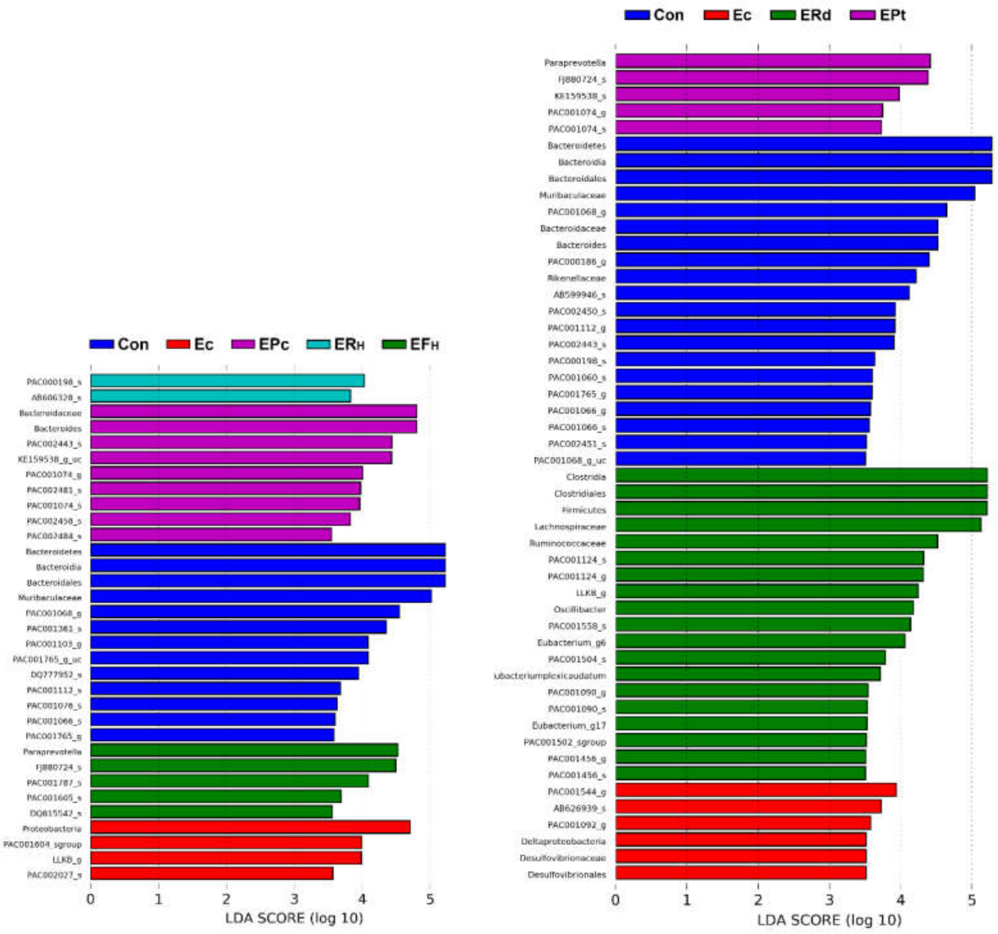

Figure S3. Effects of red ginseng (RG), fermented RG (fRG), and their constituents ginsenoside Rd and protopanaxatriol on gut microbiota composition in mice with *Escherichia coli* K1-induced anxiety/depression. (A) Effects of red ginseng (RG) and fermented RG (fRG): NC (blue), Ec (red), Pc (purple), ER_H (blue-green), and EF_H (green) groups. (B) Effects of ginsenoside Rd and protopanaxatriol: NC (blue), Ec (red), ERd (green), and EPt (purple) groups. Test agents treated in each group are indicated in Supplement Tables S2 and

S4. The described strains were analyzed to the Linear Discriminant Analysis (LDA) along with effect size measurement (LEfSE) in Galaxy (http://huttenhower.sph.harvard.edu/galaxy/). It was used to discriminate significant differentially strains at each taxon level. The threshold logarithmic score set at 3.5 and ranked. Bacterial strains were described based on 16SrRNA sequencing data.

[Figure S4]

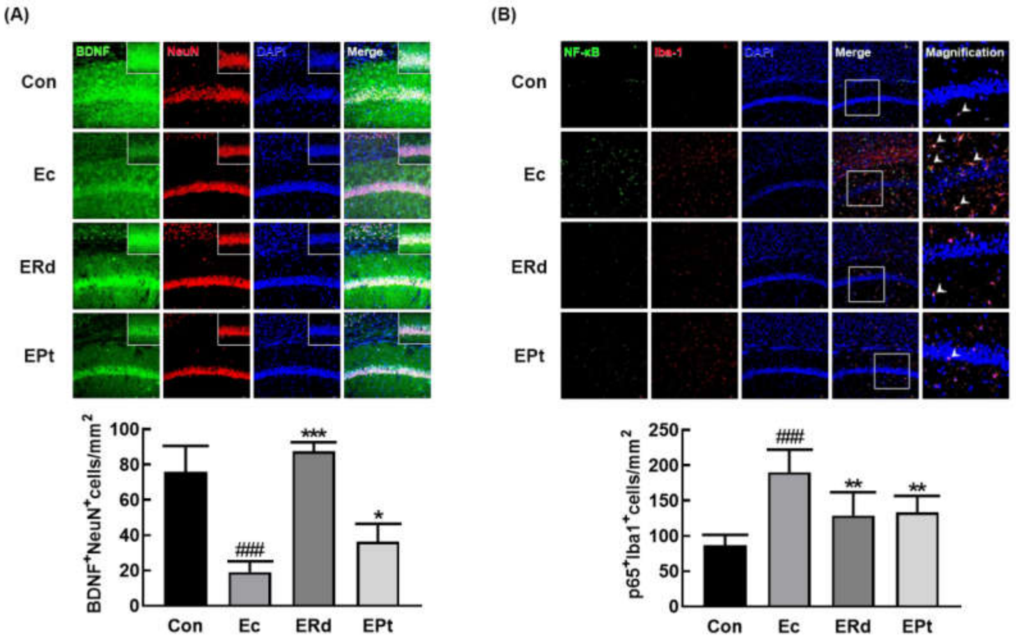

Figure S4. Effects of ginsenoside Rd and protopanaxatriol on the infiltration of $BDNF^{+}/NeuN^{+}$ (H) and NF-κB^{+}/$Iba1^{+}$ cells (I) into the CA1 region of hippocampus of *Escherichia coli* K1-exposed mice. Mice were exposed to *Escherichia coli* K1 (EC, 1×10^{9} CFU/mouse/day) and thereafter test agents (Ec, vehicle [saline]; ERd, 5 mg/kg/day of ginsenoside Rd; EPt, 5 mg/kg/day of protopanaxatriol) were gavaged daily for 5 days. Normal control group (Con), not exposed to EC, was treated with saline instead of test

agents. Data values were indicated as means ± SD (n = 6). $^{\#\#\#}p < 0.05$ vs. Con group. $^{*}p < 0.05$ vs. Ec group. $^{**}p < 0.01$ vs. Ec group. $^{***}p < 0.001$ vs. Ec group.

[Table S1]

Table S1. *p*-values in all experiments

**Figure 1.**					
	Group	*p*-value (vs Is group)		Group	*p*-value (vs Is group)
(A) (a) OT (%)	Con	0.000021	(A) (b) OE (%)	Con	0.003631
	IPc	0.002789		IPc	0.042224
	IR_L	0.001527		IR_L	0.027234
	IR_H	0.000036		IR_H	0.002189
	IF_L	0.006663		IF_L	0.042472
	IF_H	0.000428		IF_H	0.006682
(B) TL (%)	Con	0.023586	(C) Immobility (%)	Con	0.031370
	IPc	0.030334		IPc	0.000016
	IR_L	0.025779		IR_L	0.000058
	IR_H	0.017290		IR_H	0.000030
	IF_L	0.034794		IF_L	0.000137
	IF_H	0.006646		IF_H	0.000003
(D) Fold change (BDNF/β-actin)	Con	0.022107	(D) Fold change (p-p65/p65)	Con	0.000660
	IPc	0.000002		IPc	0.002177
	IR_H	3.3842E-7		IR_H	0.016019
	IF_H	6.5008E-8		IF_H	0.000262
(E) CORT (ng/mL)	Con	0.000013	(F) IL-6 (pg/mL)	Con	0.000950
	IPc	3.5837E-7		IPc	0.031020
	IR_L	0.118971		IR_L	0.008057
	IR_H	0.000004		IR_H	0.000277
	IF_L	0.099370		IF_L	0.001962
	IF_H	2.0853E-7		IF_H	0.000167
(G) Colon length (cm)	Con	0.013908	(H) MPO activity (μU/mg)	Con	0.024960
	IPc	0.021697		IPc	0.025864
	IR_L	0.013731		IR_L	0.016624
	IR_H	0.007046		IR_H	0.022707
	IF_L	0.041858		IF_L	0.023531

	IF_H	0.003649		IF_H	0.012965
(I) TNF-α (pg/mg)	Con	0.000327	(J) IL-6 (pg/mg)	Con	0.001297
	IPc	0.020404		IPc	0.000535
	IR_L	0.000054		IR_L	0.293993
	IR_H	0.000425		IR_H	0.035441
	IF_L	0.000028		IF_L	0.397433
	IF_H	0.000002		IF_H	0.034869
(K) Fold change (p-p65/p65)	Con	0.008829			
	IPc	0.000503			
	IR_H	0.023051			
	IF_H	0.016322			
**Figure 2.**					
	Group	*p*-value (vs Ec group)		Group	*p*-value (vs Ec group)
(A) (a) OT (%)	Con	0.000071	(A) (b) OE (%)	Con	0.004252
	EPc	0.042920		EPc	0.027787
	ER_L	0.378629		ER_L	0.024371
	ER_H	0.026774		ER_H	0.010082
	EF_L	0.012531		EF_L	0.012852
	EF_H	0.003682		EF_H	0.004064
(B) TL (%)	Con	0.029468	(C) Immobility (%)	Con	0.026514
	EPc	0.996496		EPc	0.043568
	ER_L	0.999886		ER_L	0.651567
	ER_H	0.041576		ER_H	0.033086
	EF_L	0.944698		EF_L	0.003998
	EF_H	0.033845		EF_H	0.000785
(D) Immobility (%)	Con	0.016511	(E) CORT (ng/mL)	Con	0.000023
	EPc	0.029279		EPc	0.000247
	ER_L	0.305523		ER_L	0.000051
	ER_H	0.043954		ER_H	0.000009
	EF_L	0.002538		EF_L	0.000038
	EF_H	0.001722		EF_H	2.8117E-8
(F) IL-6 (pg/mL)	Con	0.016188	(G) Fold change (p-CREB/CREB)	Con	0.012026
	EPc	0.034815		EPc	0.003097
	ER_L	0.085350		ER_L	0.262555
	ER_H	0.045626		ER_H	0.000323
	EF_L	0.035394		EF_L	0.010061
	EF_H	0.030909		EF_H	0.008080
(G) Fold change (BDNF/β-actin)	Con	0.027583	(G) Fold change (p-p65/p65)	Con	0.000271
	EPc	0.000113		EPc	0.000310
	ER_L	0.336057		ER_L	0.278920
	ER_H	0.011595		ER_H	0.004157

	EF_L	0.011366		EF_L	0.004184
	EF_H	0.000706		EF_H	0.000021
(H) BDNF$^+$NeuN$^+$ cells/mm^2	Con	2.0008E-8	(I) p65$^+$Iba1$^+$ cells/mm^2	Con	3.8779E-9
	EPc	1.5813E-7		EPc	0.000016
	ER_L	0.990575		ER_L	0.091286
	ER_H	0.000036		ER_H	0.000001
	EF_L	0.000310		EF_L	1.4596E-8
	EF_H	0.000005		EF_H	2.665E-9
**Figure 3.**					
	Group	*p*-value (vs Ec group)		Group	*p*-value (vs Ec group)
(A) Colon length (cm)	Con	0.035045	(B) MPO activity (μU/mg)	Con	0.041277
	EPc	0.166392		EPc	0.039069
	ER_L	0.863562		ER_L	0.966864
	ER_H	0.441059		ER_H	0.943107
	EF_L	0.521660		EF_L	0.598657
	EF_H	0.029623		EF_H	0.035169
(C) TNF-α (pg/mg)	Con	0.013775	(D) IL-6 (pg/mg)	Con	0.046968
	EPc	0.018106		EPc	0.037173
	ER_L	0.865282		ER_L	0.871550
	ER_H	0.943871		ER_H	0.648004
	EF_L	0.547990		EF_L	0.133912
	EF_H	0.003541		EF_H	0.049751
(E) Fold change (p-p65/p65)	Con	0.000206	(F) p65$^+$CD11c$^+$ cells/mm^2	Con	6.0138E-7
	EPc	0.049383		EPc	0.040839
	ER_L	0.506996		ER_L	0.052565
	ER_H	0.033133		ER_H	0.008426
	EF_L	0.004279		EF_L	0.000321
	EF_H	0.000037		EF_H	0.000599
(G) OTUs	Con	0.927274	(H) Shannon	Con	0.975357
	EPc	0.999440		EPc	0.993348
	ER_H	0.854573		ER_H	0.999869
	EF_H	0.941668		EF_H	0.996571
**Figure 4.**					
	Group	*p*-value (vs Ec group)		Group	*p*-value (vs Ec group)
(A) (a) OT (%)	Con	0.025918	(A) (b) OE (%)	Con	0.004450
	ERd	0.003042		ERd	0.017678
	EPt	0.687474		EPt	0.601490
(B) TL (%)	Con	0.012279	(C) Immobility (%)	Con	0.000005
	ERd	0.001361		ERd	0.004311
	EPt	0.014292		EPt	0.023807

(D) Immobility (%)	Con	0.006410	(E) CORT (ng/mL)	Con	0.007274
	ERd	0.001015		ERd	0.016682
	EPt	0.035407		EPt	0.027051
(F) IL-6 (pg/mL)	Con	0.012979	(G) Fold change (p-CREB/CREB)	Con	0.045136
	ERd	0.026820		ERd	0.019262
	EPt	0.039161		EPt	0.016529
(G) Fold change (BDNF/β-actin)	Con	0.024464	(G) Fold change (p-p65/p65)	Con	0.008601
	ERd	0.044789		ERd	0.000359
	EPt	0.003803		EPt	0.001153
(H) $BDNF^+NeuN^+$ cells/mm^2	Con	0.008919	(I) $p65^+Iba1^+$ cells/mm	Con	5.287E-8
	ERd	0.014866		ERd	0.000035
	EPt	0.016455		EPt	0.000134
**Figure 5.**					
	Group	*p*-value (vs Ec group)		Group	*p*-value (vs Ec group)
(A) Colon length (cm)	Con	0.000347	(B) MPO activity (μU/mg)	Con	0.002956
	ERd	0.022593		ERd	0.006946
	EPt	0.027028		EPt	0.005522
(C) TNF-α (pg/mg)	Con	0.001644	(D) IL-6 (pg/mg)	Con	0.031687
	ERd	0.021699		ERd	0.006143
	EPt	0.008708		EPt	0.012741
(E) Fold change (p-p65/p65)	Con	0.000316	(F) $p65^+CD11c^+$ cells/mm^2	Con	0.000082
	ERd	0.003074		ERd	0.009001
	EPt	0.008621		EPt	0.011539
(G) OTUs	Con	0.854857	(H) Shannon	Con	0.828057
	ERd	0.037527		ERd	0.157862
	EPt	0.014158		EPt	0.985737
**Supplementary Figure S1.**					
	Group	*p*-value (vs Is group)		Group	*p*-value (vs Is group)
(A) (a) OT (%)	Con	0.001296	(A) (b) OE (%)	Con	0.034588
	IR_{10}	0.010489		IR_{10}	0.012195
	IR_{25}	0.000136		IR_{25}	0.000222
	IR_{50}	0.807492		IR_{50}	0.999565
	IF_{10}	0.045110		IF_{10}	0.025645
	IF_{25}	0.000049		IF_{25}	0.000817
	IF_{50}	0.693413		IF_{50}	0.045091
(B) Immobility (%)	Con	0.049881			
	IR_{10}	0.001310			
	IR_{25}	0.000048			
	IR_{50}	1.000000			
	IF_{10}	0.000178			

	IF_{25}	0.000002			
	IF_{50}	0.999799			
**Supplementary Figure S2.**					
	Group	*p*-value (vs Ec group)		Group	*p*-value (vs Ec group)
(A) BDNF$^+$NeuN$^+$ cells/mm^2	Con	0.002720	(B) p65$^+$Iba1$^+$ cells/mm^2	Con	3.2095E-8
	EPc	0.010400		EPc	0.000001
	ER_L	0.993193		ER_L	0.002022
	ER_H	0.016968		ER_H	0.000242
	EF_L	0.047783		EF_L	0.000205
	EF_H	0.000248		EF_H	0.000003
**Supplementary Figure S4.**					
(A) BDNF$^+$NeuN$^+$ cells/mm^2	Con	2.2196E-8	(B) p65$^+$Iba1$^+$ cells/mm^2	Con	0.000002
	ERd	8.7717E-10		ERd	0.002578
	EPt	0.033072		EPt	0.007500

[Table S2]

Table S2. Effects of red ginseng (RG) and fermented RG (fRG) on the gut microbiota composition at the family level in mice with *Escherichia coli*-induced anxiety/depression

	**Average**					**Standard Deviation**				
**Taxon Name**	**Con**	**Ec**	**EPc**	**ER_H**	**EF_H**	**Con**	**Ec**	**EPc**	**ER_H**	**EF_H**
AC160630_f	0.53	0.56	0.62	0.39	0.20	0.20	0.51	0.29	0.18	0.13
Bacteroidaceae	9.46	3.53$^{\#}$	16.76*	10.85	6.29*	4.65	1.56	9.84	7.15	1.77
Christensenellaceae	1.04	0.59	0.50	0.68	0.48	1.48	0.62	0.59	0.26	0.34
Desulfovibrionaceae	0.28	0.86	0.56	0.78	0.48	0.04	0.58	0.27	0.76	0.26
Enterobacteriaceae	0.95	5.94	0.16	0.01	0.00	1.21	12.37	0.33	0.01	0.00
FR888536_f	0.35	0.41	0.65	0.61	1.45	0.25	0.37	0.51	1.06	2.14
Helicobacteraceae	1.94	3.73	1.38*	2.62	3.02	0.68	1.96	0.55	1.71	1.35
Lachnospiraceae	14.79	30.88$^{\#}$	16.40	22.74	18.41	4.32	14.45	6.46	14.41	10.24
Lactobacillaceae	0.56	0.51	0.36	0.21	0.20	0.31	0.53	0.23	0.09	0.14
Muribaculaceae	43.38	21.31$^{\#}$	31.62*	29.90	33.26	4.32	5.79	7.37	13.27	11.06
Odoribacteraceae	1.06	0.17	0.71	1.63*	0.24	0.92	0.08	0.87	1.40	0.32
Porphyromonadaceae	0.76	0.54	0.82	0.80	0.68	0.39	0.38	0.20	0.44	0.23
Prevotellaceae	11.03	13.86	16.69	14.39	19.28	7.51	10.89	10.73	6.40	8.91
Rikenellaceae	5.52	3.11$^{\#}$	4.57	5.50	5.07	1.67	1.58	1.73	3.00	1.71
Ruminococcaceae	6.16	11.20$^{\#}$	6.14	6.85	8.06	2.63	3.75	4.26	4.11	2.66

Mice were exposed to *Escherichia coli* (1×10^9 CFU/mouse/day) and test agents (EC, vehicle [saline]; ER_L, 10 mg/kg/day of RG; ER_H, 25 mg/kg/day of RG; EF_L, 10 mg/kg/day of fRG; EF_H, 25 mg/kg/day of fRG) were gavaged (for vehicle, RG, and fRG) or intraperitoneally injected (for buspirone) daily for 5 days. Normal control group (Con), not exposed to EC, was treated with saline instead of test agents. *p<0.05 vs. Con group, $^{\#}p$<0.05 vs Ec group.

[Table S3]

Table S3. Effects of red ginseng (RG) and fermented RG (fRG) on the gut microbiota composition at the genus level in mice with *Escherichia coli*-induced anxiety/depression

	Average					Standard Deviation				
**Taxon Name**	**Con**	**Ec**	**EPc**	**ER_H**	**EF_H**	**Con**	**Ec**	**EPc**	**ER_H**	**EF_H**
Alistipes	5.03	2.41$^{\#}$	4.29	5.05	4.46*	1.67	1.13	1.69	2.98	1.42
Bacteroides	9.44	3.53$^{\#}$	16.73*	10.79	6.29*	4.65	1.56	9.81	7.10	1.78
Escherichia	0.94	5.87	0.16	0.01	0.00	1.19	12.21	0.33	0.01	0.00
Helicobacter	1.94	3.72	1.38*	2.62	3.02	0.68	1.95	0.55	1.71	1.35
KE159538_g	5.37	2.99	6.62	4.60	1.71	3.83	3.25	5.37	2.37	1.41
Lactobacillus	0.56	0.51	0.36	0.21	0.20	0.31	0.53	0.23	0.09	0.14
Muribaculum	3.08	1.17	1.53	1.10	1.83	1.78	0.56	0.56	0.62	0.95
Mycoplasma_g10	0.90	0.34	0.48	0.48	0.33	1.15	0.24	0.41	0.46	0.14
Odoribacter	1.05	0.16	0.71	1.62*	0.24	0.91	0.08	0.87	1.39	0.32
Oscillibacter	1.05	2.84$^{\#}$	0.97*	1.95	2.56	0.36	1.49	0.87	1.84	2.23
PAC000186_g	11.39	6.31$^{\#}$	8.42	6.73	9.31	1.10	3.00	2.43	3.63	3.76
PAC000198_g	2.85	1.58	2.06	3.86	2.48*	1.26	0.78	0.57	2.45	0.36
PAC001068_g	13.98	6.73$^{\#}$	9.22	9.49	10.74	1.29	1.64	1.95	3.18	5.44
PAC001074_g	1.19	0.29$^{\#}$	2.24*	1.88*	1.60*	0.53	0.19	0.92	1.32	0.92
PAC001091_g	1.45	5.63	1.92	0.64	0.70	1.36	10.86	1.85	0.33	0.73
PAC001512_g	1.56	1.93	1.45	1.60	2.46	1.45	1.44	1.14	1.01	1.22
PAC002367_g	0.42	1.81	0.14	1.22	2.50	0.65	2.54	0.20	2.34	3.39
Parabacteroides	0.76	0.54	0.82	0.80	0.68	0.39	0.38	0.20	0.44	0.23
Paraprevotella	2.79	0.71	3.75*	1.26	6.54*	2.32	0.80	2.55	1.44	4.54
Parasutterella	0.25	0.08$^{\#}$	0.27*	0.12	0.16	0.15	0.03	0.16	0.11	0.10

Prevotella	4.16	3.04	4.64	2.61	3.01	1.99	3.78	1.65	0.41	1.13
Prevotellaceae_uc	4.06	10.11	8.28	9.47	9.69	6.97	7.58	10.83	8.79	5.34
Pseudoflavonifractor	2.25	3.13	2.08	2.50	2.03	0.59	1.82	0.70	1.70	1.36
Ruminococcus	1.16	1.76	1.42	0.56	1.04	1.78	1.38	1.74	0.79	1.00

Mice were exposed to *Escherichia coli* (1×10^9 CFU/mouse/day) and test agents (EC, vehicle [saline]; ER_L, 10 mg/kg/day of RG; ER_H, 25 mg/kg/day of RG; EF_L, 10 mg/kg/day of fRG; EF_H, 25 mg/kg/day of fRG) were gavaged (for vehicle, RG, and fRG) or intraperitoneally injected (for buspirone) daily for 5 days. Normal control group (Con), not exposed to EC, was treated with saline instead of test agents. *$p<0.05$ vs. Con group, $^{\#}p<0.05$ vs Ec group.

[Table S4]

Table S4. Effects of ginsenoside Rd and protoanaxatriol on the gut microbiota composition at the family level in mice with *Escherichia coli*-induced anxiety/depression

	**Average**				**Standard Deviation**			
**Taxon Name**	**Con**	**Ec**	**ERd**	**EPt**	**Con**	**Ec**	**ERd**	**EPt**
AC160630_f	0.53	0.56	0.28	0.94	0.20	0.51	0.12	0.38
Bacteroidaceae	9.46	3.53#	2.45	6.03*	4.65	1.56	1.14	1.83
Christensenellaceae	1.04	0.59	0.36	0.23	1.48	0.62	0.12	0.12
Desulfovibrionaceae	0.28	0.86	0.55	0.80	0.04	0.58	0.13	0.22
Enterobacteriaceae	0.95	5.94	1.14	1.22	0.03	1.71	1.00	1.11
FR888536_f	0.35	0.41	0.03	0.00	1.21	12.37	0.04	0.00
Helicobacteraceae	1.94	3.73	7.75	6.99	0.68	1.96	4.65	5.32
Lachnospiraceae	14.79	30.88#	39.22	28.57	4.32	14.45	9.29	11.54
Lactobacillaceae	0.56	0.51	0.31	0.68	0.31	0.53	0.08	0.59
Muribaculaceae	43.38	21.31#	20.53	26.88	4.32	5.79	1.90	10.54
Odoribacteraceae	1.06	0.17	0.31*	0.73	0.92	0.08	0.11	0.73
Porphyromonadaceae	0.76	0.54	0.48	0.52	0.39	0.38	0.14	0.14
Prevotellaceae	11.03	13.86	11.65	12.22	7.51	10.89	11.19	3.59
Rikenellaceae	5.52	3.11#	1.96	3.65	1.67	1.58	0.85	2.30
Ruminococcaceae	6.16	11.20#	11.74	8.71	2.63	3.75	1.68	2.57

Mice were exposed to *Escherichia coli* (1×10^9 CFU/mouse/day) and test agents (EC, vehicle [saline]; ERd, 5

mg/kg/day of ginsenoside Rd; EPt, 5 mg/kg/day of protopanaxatriol) were gavaged daily for 5 days. Normal control group (Con), not exposed to EC, was treated with saline instead of test agents. *p<0.05 vs. Con group, $^{\#}p$<0.05 vs Ec group.

[Table S5]

Table S5. Effects of ginsenoside Rd and protoanaxatriol on the gut microbiota composition at the family level in mice with *Escherichia coli*-induced anxiety/depression

	**Average**				**Standard Deviation**			
**Taxon Name**	**Con**	**Ec**	**ERd**	**EPt**	**Con**	**Ec**	**ERd**	**EPt**
Alistipes	5.03	2.41#	1.64	3.31	1.67	1.13	0.77	2.16
Bacteroides	9.44	3.53#	2.44	6.03*	4.65	1.56	1.14	1.83
Escherichia	0.94	5.87	0.03	0.00	1.19	12.21	0.04	0.00
Helicobacter	1.94	3.72	7.73	6.97	0.68	1.95	4.63	5.29
KE159538_g	5.37	2.99	5.83	5.73	3.83	3.25	3.32	4.53
Lactobacillus	0.56	0.51	0.31	0.68	0.31	0.53	0.08	0.59
LLKB_g	0.16	1.94#	3.26	1.73	0.14	0.80	2.97	1.65
Muribaculum	3.08	1.17	1.43	1.64	1.78	0.56	0.66	0.68
Mycoplasma_g10	0.90	0.34	0.18	0.23	1.15	0.24	0.29	0.16
Odoribacter	1.05	0.16	0.31*	0.73	0.91	0.08	0.11	0.73
Oscillibacter	1.05	2.84#	3.74	2.24	0.36	1.49	1.29	0.89
PAC000186_g	11.39	6.31#	6.92	7.30	1.10	3.00	1.33	2.29
PAC000198_g	2.85	1.58	1.04	1.30	1.26	0.78	0.25	0.34
PAC001068_g	13.98	6.73#	5.12	7.59	1.29	1.64	0.95	3.55
PAC001091_g	1.45	5.63	3.35	3.56	1.36	10.86	3.23	4.40
PAC001112_g	2.03	0.53#	1.06	0.94	1.24	0.21	0.48	0.35
PAC001124_g	0.28	1.31	4.08	1.02	0.49	2.31	2.84	1.28
PAC002367_g	0.42	1.81	2.02	2.48	0.65	2.54	2.83	2.92
Parabacteroides	0.76	0.54	0.48	0.52	0.39	0.38	0.14	0.14
Paraprevotella	2.79	0.71	0.28	4.60	2.32	0.80	0.39	4.05
Parasutterella	0.25	0.08#	0.04	0.05	0.15	0.03	0.04	0.04
Prevotella	4.16	3.04	1.64	1.38	1.99	3.78	1.33	0.93

Prevotellaceae_uc	4.06	10.11	9.73	5.99	6.97	7.58	10.19	2.88
Pseudoflavonifractor	2.25	3.13	2.77	2.48	0.59	1.82	0.34	0.96

Mice were exposed to *Escherichia coli* (1×10^9 CFU/mouse/day) and test agents (EC, vehicle [saline]; ERd, 5 mg/kg/day of ginsenoside Rd; EPt, 5 mg/kg/day of protopanaxatriol) were gavaged daily for 5 days. Normal control group (Con), not exposed to EC, was treated with saline instead of test agents. $*p<0.05$ vs. Con group, $^{\#}p<0.05$ vs Ec group.

[Table S6]

Pharmacokinetic study of protopanaxatriol in healthy volunteers orally treated with red ginseng (RG) or Bifidobacteria-fermented RG (fRG) extracts

Pharmacokinetic study of protopanaxatriol in healthy volunteers orally treated with RG or FRG extracts was performed, as previously reported [1,2]. The healthy volunteers were orally given RG or fRG at a dose of 3 g/60kg/day and the blood samples were collected for 24 h. Subsequently, the serum concentrations of protopanaxatrol were determined using LC-MS/MS analysis. The pharmacokinetic parameters are summarized in Table S6.

Table S6. Pharmacokinetic parameters of protopanaxatriol in healthy volunteers orally treated with RG or fRG

Ginsenoside	Parameter	RG-group (n=17)	fRG-group (n=18)	p-value$^{\#}$
protopanaxatriol	C_{max} (ng/mL)	13.48 ± 13.68	26.74 ± 26.47	0.124
	AUC_t (ng·h/mL)	109.30 ± 77.29	214.76 ± 172.60	0.053
	AUC_∞ (ng·h/mL)	138.20 ± 93.35	274.50 ± 242.91	0.072
	T_{max} (h)	13.59 ± 6.29	12.00 ± 6.09	0.461

$^{\#}$p-value by Wilcoxon rank sum test

References

[1] Kim, J.K.; Jang, S.E.; Choi, M.S.; Jang, H.M.; Yoo, H.H.; Kim, D.H. Fermented red ginseng alleviates cyclophosphamide-induced immunosuppression and 2,4,6-trinitrobenzenesulfonic acid-induced colitis in mice by regulating macrophage activation and T cell differentiation. *Am J. Chi.n Med*. **2018**, 46, 1879-1897.

[2] Kim, J.K.; Choi, M.S.; Jeung, W.; Ra, Y.S.; Yoo, H.H.; Kim, D.H. *J. Ginseng Res*. **2020** in press
